# Apparent Motion Is Computed in Perceptual Coordinates

**DOI:** 10.1177/2041669520933309

**Published:** 2020-07-14

**Authors:** Jiahan Hui, Yue Wang, Peng Zhang, Peter U. Tse, Patrick Cavanagh

**Affiliations:** Department of Psychological and Brain Sciences, Dartmouth College, Hanover, New Hampshire, United States; State Key Laboratory of Brain and Cognitive Science, Institute of Biophysics, Chinese Academy of Sciences, Beijing, China; State Key Laboratory of Brain and Cognitive Science, Institute of Biophysics, Chinese Academy of Sciences, Beijing, China; College of Life Sciences, University of Chinese Academy of Sciences, Beijing, China; Department of Psychological and Brain Sciences, Dartmouth College, Hanover, New Hampshire, United States; Department of Psychological and Brain Sciences, Dartmouth College, Hanover, New Hampshire, United States; Department of Psychology, Glendon College, CVR York University, Toronto, Ontario, Canada

**Keywords:** apparent motion, motion-induced position shift, perceptual coordinates, double-drift stimulus

## Abstract

When a Gabor moves in one direction in the visual periphery while its internal texture moves in the orthogonal direction, its perceived direction can deviate from its physical direction by as much as 45° or more. Lisi et al. showed that immediate saccades go to the physical location of double-drift targets, whereas delayed saccades primarily go to their perceived locations. Here, we investigated whether the apparent motion seen from the offset of a double-drift stimulus to the onset of a later target probe originates from the perceived or physical location of the double-drift stimulus. We find that apparent motion proceeds away from the perceived position of the double-drift stimulus at all temporal delays. This suggests that apparent motion is computed in perceptual rather than retinotopic coordinates.

We rely on our perception of the world to guide our actions. However, our perception does not always reflect the physical world. Visual illusions can reveal a mismatch between a stimulus’s perceived location and the position that corresponds to its retinal location. When a Gabor patch moves in one direction in the visual periphery while its internal texture moves in the orthogonal direction (Figure 1), its perceived direction can deviate from its physical direction by as much as 45° or more (Gurnsey & Biard, 2012; Kwon et al., 2015; Shapiro et al., 2010; Tse & Hsieh, 2006). However, Lisi and Cavanagh (2015) found that saccades do not show this illusion—they target the physical location of the stimulus, not its perceived location. Here, we are interested in whether apparent motion is also immune to the double-drift illusion. Apparent motion has been described as attention-based motion, at least for large displacements (Cavanagh, 1992), and spatial attention is strongly linked to the saccade system (Awh et al., 2006), so it is plausible that apparent motion would operate in saccade coordinates rather than perceptual coordinates. Moreover, saccades target the physical location only if the stimulus is present when the saccade is being programmed and increasingly deviate toward the perceived location with longer delays between the offset of the moving Gabor and the cue to make a saccade (Massendari et al., 2018). We therefore tested the direction of apparent motion at different delays to see whether longer delays also caused a similar shift of the effective location of the offset from its physical to its perceived location.

**Figure 1.**
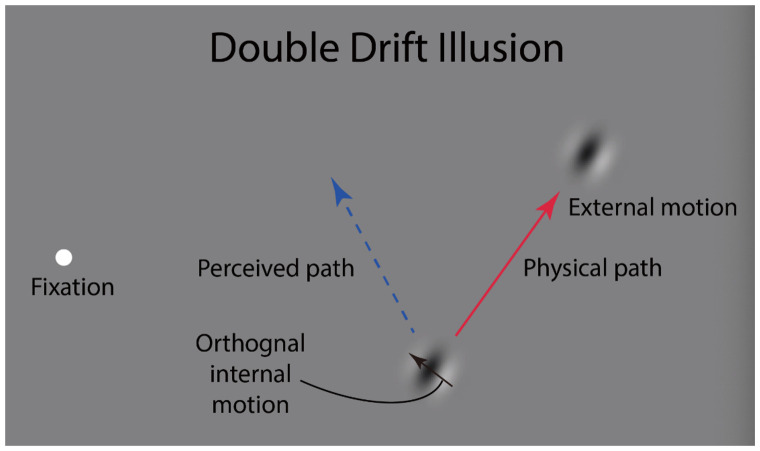
Double-Drift Stimulus. The internal motion of the Gabor is orthogonal to the physical path. The red arrow shows the physical path of the Gabor and the dotted blue line shows a typical perceived path of the Gabor when viewing the target in the periphery. See Supplemental Video 1.

**Figure 2.**
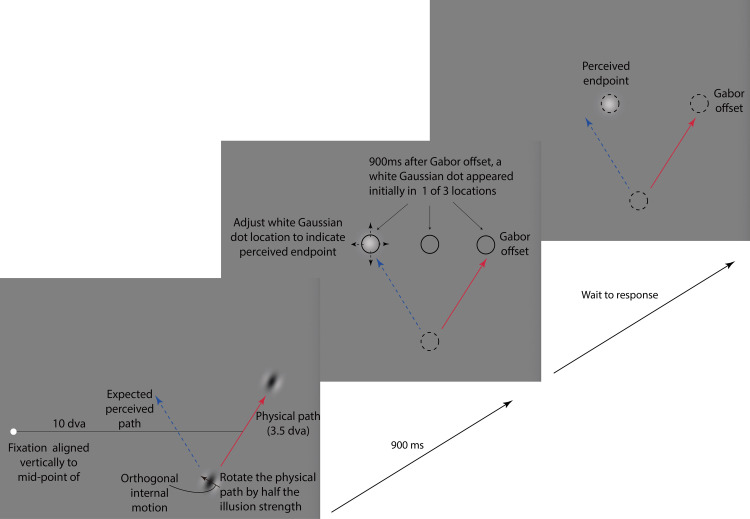
Experiment 1. While the participants kept their gaze on the fixation dot, the Gabor targetmoved while its internal grating drifted orthogonally to the Gabor’s path. After traveling for 1,750 milliseconds, the Gabor turned off and 900 milliseconds later a white Gaussian dot appeared that participants adjusted to report the perceived endpoint of the Gabor’s trajectory. See Supplemental Video 2 for a sample trial.

We examined the apparent motion from the offset of the double-drift target to a briefly flashed test. The perceived location of the double-drift target can be quite far from its physical location and we evaluated whether the motion appears to originate from the perceived endpoint or from the physical location. To do so, we first determined where the endpoint was perceived (Experiment 1). A Gabor traveled for 1,750 milliseconds and then turned off. After 900 milliseconds, a probe appeared and participants adjusted its location to indicate where they saw the endpoint of the double-drift motion path. Because the onset of the white Gabor might disrupt the illusion, we delayed its onset to 900 milliseconds after Gabor offset. This interval was long enough so that there was no apparent motion between the offset of the double-drift target and the probe, making these judgments a valid index of the location of the perceived offset. We then used this measure of the perceived endpoint to evaluate the apparent motion in Experiment 2.

## Method

### Participants

Ten observers, whose ages range from 20 to 38 years participated in the experiment (seven women including one author; mean age = 24.4 years, standard deviation = 1.55 years). All observers reported having normal or corrected-to-normal vision. All subjects provided written informed consent before the experiments. The institutional review boards of the Institute of Biophysics and of Dartmouth College approved the experimental protocols.

### Apparatus

In all the experiments, participants sat in a dark and quiet room. Stimuli were generated using MATLAB R2018 and PsychToolbox-3. An Apple computer running MATLAB (Mathworks) with the Psychophysics controlled the stimulus presentation and response collection. Stimuli were displayed on a 16″ nesoJXC FS210A cathode ray tube (CRT) monitor placed 60 cm from the participant with their head stabilized on a chinrest.

### Stimulus

The stimulus used for all experiments was a Gabor patch (a sinusoidal grating within a Gaussian envelope) with a spatial frequency of 2 cycles/dva and 100% contrast. The standard deviation of the Gaussian envelope was 0.1 dva (Figure 1). In each trial, the Gabor patch moved along a linear path of length 3.5 dva for 1,750 milliseconds with a speed of 2 dva/second (external motion). The sinusoidal grating had the same orientation as the motion path and drifted orthogonally to the direction of the external motion with a temporal frequency of 3 Hz (internal motion). A white fixation point (0.3 dva) was located at 3 dva horizontal to the left of the screen center. The midpoint of the Gabor path was aligned vertically to the fixation point and the starting point of the path was 10 dva horizontally to the right of the fixation.

### Procedure

#### Pre-experiment

Before the main experiments, we first measured each participant’s illusion strength for the double-drift stimulus. The same double-drift Gabor used in the main experiments was presented traveling at various physical directions from upward to the left to upward to the right, and participants were asked to report whether the perceived trajectory was tilted leftward or rightward from vertical. For each participant, an adaptive staircase procedure was used to find the physical direction that appeared vertical—where the path was judged equally likely as tilted leftward or rightward. Each participant completed 50 adjustment trials for each direction, for a total of 100 trials with two conditions, upward to the left or upward to the right, randomized and counterbalanced across the trials. The results for each direction were then averaged to determine each participant’s illusion strength. The illusion strength—the deviation from vertical that was required for the path to appear vertical—was then used to orient the physical path of the Gabor in Experiments 1 and 2 as described later. The mean value of the illusory change in direction was 46.32° ± 2.67° with a range of 17.5° to 61.9° across the 10 participants.

#### Experiment 1

The double-drift targets were identical to those used in the pre-experiment (with the same speed of external and internal motion). The physical path was tilted leftward or rightward from vertical by half of the illusion strength measured for each participant so that, ideally, the perceived path would be tilted symmetrically away from vertical on the other side (Figure 1). The Gabor’s trajectory lasted 1,750 milliseconds, and then, 900 milliseconds after its offset, a movable Gaussian white dot appeared that participants adjusted to mark where they saw the Gabor disappear (Supplementary Video 2). The initial location of the white Gaussian dot and the internal motion direction of the double-drift stimulus were randomly assigned in each trial so that observers could not develop any strategies for judging the location of the endpoint of the target object. Each experiment had six blocks and each block contained 24 trials. The trials alternated randomly between left and right tilt to cancel out any absolute response bias. For example, there is a clear bias in the experiment to favor responses nearer to the fovea but as the direction of the illusion was balanced toward and away from the fovea, the bias was canceled.

#### Experiment 2

The Gaussian white dot was flashed after the offset of the Gabor target at eight different delays from 0 to 350 milliseconds, and at 3.5 dva vertically above the endpoint. The Gabor target was identical to that used in Experiment 1 and the Gaussian dot was the same as well except for its timing and position. The task of the participants was to judge the direction of the motion or displacement they saw between the offset of the Gabor and the onset of the Gaussian flash. At 900 milliseconds after the offset of the Gaussian dot, an adjustable line appeared. One end was anchored at the location of the white flash, while the other end could be adjusted to match the point from which the motion or displacement appeared to originate. The adjusted angle of the line was recorded. Each participant performed 192 trials divided into six blocks. Trials with left and right path orientations and eight delays were randomly interleaved and counterbalanced.

## Experiment 1: The Location of the Perceived Endpoint of the Gabor’s Motion Path

### Results

**Figure 3.**
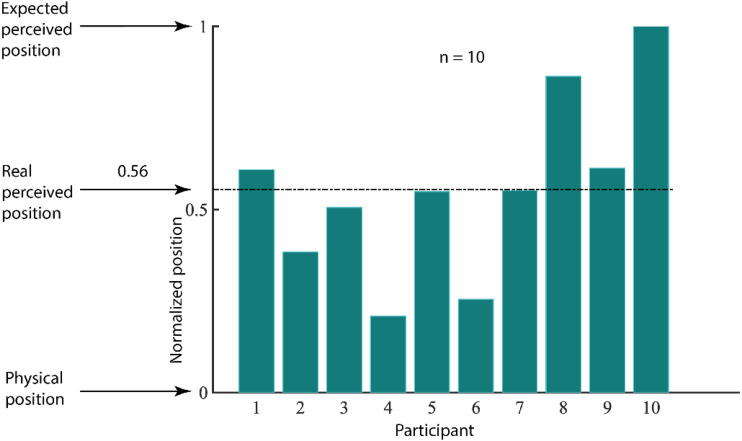
The perceived Endpoint for Individual Participants Is Plotted as a Proportion of Distance From the Physical Offset to Their Expected Perceived Endpoint (An Equal Distance From Vertical on the Other Side of the Physical Endpoint). The average normalized position is 0.56 ± 0.07 which is indicated by the black-dotted line on the graph.

The results are shown in Figure 3 as a proportion of distance to the expected perceived endpoint. The distance of the actual perceived endpoint was on average only about 50% of its expected location based on the original measure of illusory direction. This shortfall highlights the difference between taking direction measures of the illusion (as in our pre-experiment) and positional measures. It is likely that during the 1,750 milliseconds of motion, the illusion may have reset, overshooting some limit to its maximum value and returning to its physical location where the illusory shifts would begin accumulating again. These resets would reduce the final offset at the endpoint. The direction measure may have been based more on the instantaneous direction rather than an overall vector joining the start point to the endpoint. The illusion resets ('t Hart et al., 2019; Liu, Tse, et al., 2019) will change the perceived path without changing the instantaneous direction (except right at the reset where the position jumps back to the physical path). Despite the endpoint being less distant from the physical endpoint than expected, it is still a substantial distance away (55.51% of the distance from the physical endpoint to the expected perceptual endpoint if there had been no resets) so that our next test of the direction of apparent motion from the endpoint still has diagnostic power.

**Figure 4.**
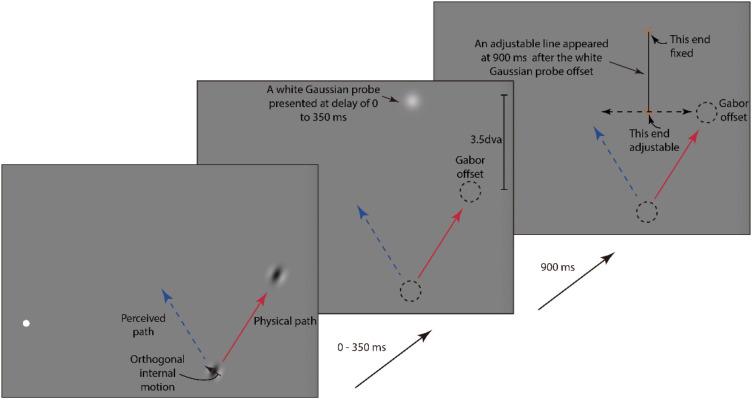
Experiment 2. The Gabor target traveled for 1,750 milliseconds then turned off. After one of the eight possible delays (0 milliseconds, 50 milliseconds, 100 milliseconds, 150 milliseconds, 200 milliseconds, 250 milliseconds, 300 milliseconds, and 350 milliseconds), a white Gaussian dot probe flashed above the start point of Gabor for 17 milliseconds. Following 900 milliseconds of blank screen, an adjustable line appeared, one end was anchored at the location of the white flash, while the other end could be moved left or right. Participants reported the direction of apparent motion or displacement by adjusting the line. See Supplemental Video 3 for a sample trial.

## Experiment 2: Delay Effects

### Results

**Figure 5.**
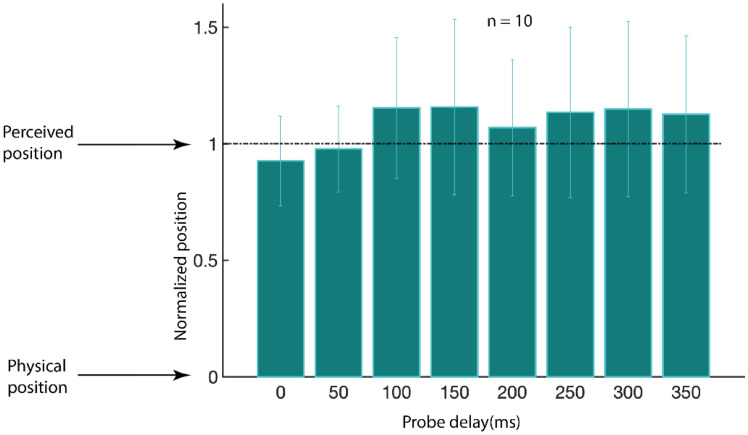
Perceived Location of the Point Where the Apparent Motion or Displacement Originated Normalized by the Perceived Location of the Gabor Offset From Experiment 1, Plotted as a Function of the Delay. Error bars are ±1 standard error. On the vertical axis, 0 means the apparent motion or displacement originated from the physical location of the Gabor’s offset and 1 means it originated from the perceived position of the offset.

The effective offset location of the Gabor corresponding to that participant’s settings is plotted in Figure 5, normalized by the distance to the perceived endpoint taken from each participant’s results from Experiment 1. Overall, the apparent motion or displacement appeared to originate from a location quite near the perceived endpoint as measured in Experiment 1 (mean location, 1.09 ± 0.03 of the distance between the physical and perceived endpoints). This was not significantly different from an origination point at the perceived offset location from Experiment 1, *t*(9) < −0.38 , *p*s > .60. Moreover, one-way repeated-measures analyses of variance were performed to compare the effect of different delays, and no effect of delay was seen, *F*(1,9) = 0.77, *p* = .402. These results suggest that apparent motion begins from the perceived position of the double-drift stimulus at all temporal delays tested.

## Summary and Discussion

Apparent motion from the offset of a double-drift stimulus to the onset of a later target probe appears to originate from the perceived location of the double-drift stimulus rather than from its physical location at all temporal offsets tested. This result is unlike that for saccades which are directed to the physical location of the Gabor target at the shortest delays (Lisi & Cavanagh, 2015) but were increasingly driven to the perceived location with longer delays (Massendari et al., 2018).

In Experiment 2, the delays between the offset of the double drift and the onset of the white dot ranged from 0 to 350 milliseconds, and we might ask whether the longer delay conditions produced impressions of apparent motion or only displacement. If they produced only an impression of displacement, then the judgments are quite similar to those of the pre-experiment where the white dot was presented after 900 milliseconds and participants adjusted it to match the perceived location of the double-drift offset. In this case, it would be unsurprising that the judgments for the *apparent motion* conditions of Experiment 2 matched the perceived location judgments in Experiment 1. However, it is the results at short delays that are the most critical test of whether apparent motion is based on perceptual or physical coordinates, and here we see that it is the perceived location that dominates. We refer the reader to Supplementary Video 3 to get a sense of the motion impressions produced by the different delays between the offset and the test dot. At the shorter delays, there is a clear sense of motion from the Gabor endpoint to the test flash. The longer delays (350 milliseconds) approach the maximum temporal delay for the experience of apparent motion (300 milliseconds, Neuhaus, 1930; at least 350 milliseconds, Kahneman & Wolman, 1970; 500 milliseconds, Mather, 1988; more than 500 milliseconds, Caelli & Finlay, 1981) so it is possible that some participants were not judging motion direction but displacement direction on some of the long-delay trials.Nevertheless, the results show that the settings at short delays, where the motion was most convincing, matched those at longer delays where there may have been a mix of motion and displacement judgments, and that all of them favored the perceived location.

A recent functional Magnetic Resonance Imaging (fMRI) experiment (Liu, Yu, et al., 2019) examined where in the brain the representation of the perceived path of the double-drift stimulus diverged from the bottom-up physical path. Surprisingly, this happened beyond the visual system, in the frontal lobes among other areas. Early visual areas encoded the independent motion vectors as well as combined local motion signals integrated over short durations, but only higher order areas outside of the visual system accumulated these signals over the long durations that create the illusory perceptual path. The perceived locations of the double-drift offset that drives the apparent motion would therefore also emerge in high-level areas and the computation of the apparent motion path from the perceived offset location must also occur there.

Importantly, fMRI results showed that these high-level representations of location for the double-drift stimulus do not feedback to early visual areas—there was no evidence of the perceived double-drift path within the visual cortical areas (Liu, Yu, et al., 2019). This suggests that the computation of the apparent motion path cannot arise in early visual areas where there is no representation of the perceived location of the double-drift offset—a location that differs from the physical location by on average, 1.4° of visual angle. This absence of top-down feedback of the perceived path contrasts sharply with the results for apparent motion where fMRI shows the construction of a filled-in representation of the motion path in early visual areas (Muckli et al., 2005). These two results appear to be in conflict: The early cortical representation of the double-drift motion is driven solely by bottom-up signals whereas that for apparent motion captures high-level filling-in along the perceived path. The explanation may be that the feedback from higher levels for apparent motion does go to the perceived locations of the motion path, whereas the feedback for the double drift does not (Liu, Yu, et al., 2019). If we assume that attention is the source of these downward projections to early visual areas, this means that attention is immune to the double-drift illusion (Liu, Yu, et al., 2019), as saccades are, whereas attention does follow the apparent motion path (e.g., Shioiri et al., 2002). This suggests that an fMRI study of the apparent motion path for the stimuli in the experiments here should reveal a large gap between the beginning of the apparent motion path (following the perceived locations) and the end of the double-drift path (at the physical location).

Whatever the outcome of such an experiment, the results here show that apparent motion from the offset of a double-drift stimulus originates from the double-drift's perceived rather than physical location at all temporal offsets. This suggests that the computation of apparent motion must occur at very high levels of the processing hierarchy, quite likely outside the visual system where the perceived location of the double-drift first emerges (Liu, Yu, et al., 2019).

An additional point arises from an enduring, general paradox about apparent motion. How is it that we see motion from the first target toward the second even though this motion cannot emerge until the appearance of the second target? One possibility is that visual information is stored in a preconscious buffer and percepts only emerge after an analysis that encompasses the events within the time span of the buffer. In this case, the position of the first target is still available and not yet consciously experienced when the second target appears and triggers the motion. For this to be consistent with the data on apparent motion, the buffer, and the delay before percepts enter awareness, would have to be as much as 500 milliseconds, the time span over which apparent motion is seen (e.g., Caelli & Finlay, 1981). The existence of a preconscious storage is plausible because we are not aware of the first target being static during the delay up to the appearance of the second. On the other hand, Sergent et al. (2013) reported that an exogenous attention cue presented 400 milliseconds after the target presentation increased its subjective visibility, a phenomenon they called retro-perception. These authors suggested that the cue triggers changes in the outcome that overwrites previous experience. Whether perception is delayed to accommodate the duration of a preconscious buffer or the final percept can rewrite our experience (or our memory of the experience), the point here is that the construction of the motion, perhaps in a preconscious buffer, operates in perceptual not physical coordinates.

## Supplementary Material

Supplemental Video 1

Supplemental Video 2

Supplemental Video 3
